# Report of March Meeting of the Odontographic Society of Chicago

**Published:** 1904-04

**Authors:** 


### REPORT OF MARCH MEETING OF THE ODONTOGRAPHIC SOCIETY OF CHICAGO.

The essayist of the evening was Dr. W. H. G. Logan, of Chicago, who read a paper on "The Diagnosis and Treatment of Fractures of the Lower Maxilla."

Dr. Logan referred briefly to the various methods and appliances used in reducing a fracture of the lower jaw, and illustrated same by means of lantern slides.

The fracture usually occurs in the weakest portion of the bone which is in the region of the cuspid tooth. Ordinarily the fragments are displaced by the action of the muscles which makes the diagnosis simple. Occasionally, however, a complete fracture exists without any displacement of the fragments. This condition can be diagnosed by placing the index finger on the lingual surface of the supposed fractured jaw, and with the other hand exert pressure on the teeth, holding the ear close to the face, in case of fracture, crepitation can be heard.

The essayist called attention to the fact that the application of bandages, for the purpose of holding the fragments in position, had become obsolete. He advised wearing the lower teeth to the upper, after the method of Dr. Gilmer, of Chicago, in those cases where the teeth in either jaw permits of such a procedure. Other cases can be treated by the proper adjustment of splints, made by taking a modeling compound impression of either jaw and obtaining models; a V-shaped place, in line of fracture, is cut in the model of the lower jaw which permits readjusting the occlusion of the lower to the upper model; now fill the V-shaped place with soft plaster, when a cast can be made from the lower model and a splint swaged of aluminum or gold. A wire should be twisted around a tooth on either side of fracture, and after the splint is cemented to place, the end can be twisted together over the splint, thus doubly securing same.

The discussion was opened by Dr. A. D. Black who displayed an intimate knowledge of the anatomy of the head. He called attention to at least nine sets of muscles which are active in the displacement of the fragments. In regard to bandages, he agreed with the essayist, but favored a splint made of vulcanite rather than of metal. It is easier constructed, and when waxed up on an articulator, the upper model can be pressed down into the wax, making impressions of the upper teeth which aid in mastication after the adjustment of the splint.

Dr. T. L. Gilmore followed and spoke very encouragingly to young men who were taking up specialties and complimented both Drs. Logan and Black upon the work they are doing. Like Dr. Black he favored the vulcanite splint, and said that a bandage was often serviceable as a means of relieving the pain while the splint was being constructed.

Dr. T. W. Brophy joined the discussion and favored the metal splint because it could be made less bulky and more hy-
He said a properly constructed vulcanite gienic. He said a properly constructed vulcanite splint was often serviceable, however, in reducing a fracture in an edentulous mouth. Some cases cannot be successfully treated without wiring the fragments, using a heavy silver wire for the purpose.

Interesting cases from practice were related by Drs. C. E. Bently, W. H. Taggart and G. V. Black.—*J. P. B.*

